# Comprehensive review on Alzheimer's disease: From the posttranslational modifications of Tau to corresponding treatments

**DOI:** 10.1002/ibra.12176

**Published:** 2024-09-16

**Authors:** Xin Li, Zhisheng Ba, Juan Huang, Jianhua Chen, Jinyu Jiang, Nanqu Huang, Yong Luo

**Affiliations:** ^1^ Department of Neurology The Third Affiliated Hospital of Zunyi Medical University (The First People's Hospital of Zunyi) Zunyi China; ^2^ National Drug Clinical Trial Institution The Third Affiliated Hospital of Zunyi Medical University (The First People's Hospital of Zunyi) Zunyi China; ^3^ Key Laboratory of Basic Pharmacology and Joint International Research Laboratory of Ethnomedicine of Ministry of Education Zunyi Medical University Zunyi China; ^4^ Department of medicine Guizhou Aerospace Hospital Zunyi China

**Keywords:** Alzheimer's disease, post‐translational modifications, Tau, treatments

## Abstract

Alzheimer's disease (AD) is a neurodegenerative disease, which is mainly characterized by the abnormal deposition of β‐amyloid peptide (Aβ) and Tau. Since Tau aggregation is more closely associated with synaptic loss, neurodegeneration, and cognitive decline than Aβ, the correlation between Tau and cognitive function in AD has gradually gained attention. The posttranslational modifications (PTMs) of Tau are key factors contributing to its pathological changes, which include phosphorylation, acetylation, ubiquitination, glycosylation, glycation, small ubiquitin‐like modifier mediated modification (SUMOylation), methylation, succinylation, etc. These modifications change the structure of Tau, regulating Tau microtubule interactions, localization, degradation, and aggregation, thereby affecting its propensity to aggregate and leading to neuronal injury and cognitive impairments. Among numerous PTMs, drug development based on phosphorylation, acetylation, ubiquitination, and SUMOylation primarily involves enzymatic reactions, affecting either the phosphorylation or degradation processes of Tau. Meanwhile, methylation, glycosylation, and succinylation are associated with maintaining the structural stability of Tau. Current research is more extensive on phosphorylation, acetylation, ubiquitination, and methylation, with related drugs already developed, particularly focusing on phosphorylation and ubiquitination. In contrast, there is less research on SUMOylation, glycosylation, and succinylation, requiring further basic research, with the potential to become novel drug targets. In conclusion, this review summarized the latest research on PTMs of Tau and related drugs, highlighting the potential of targeting specific PTMs for developing novel therapeutic strategies in AD.

## Graphical abstract

Pathological Tau is one of the primary pathological features of AD. Tau aggregation is more closely associated with synaptic loss, neurodegeneration, and cognitive decline than Aβ in Alzheimer's disease (AD). Tau can undergo various posttranslational modifications (PTMs), including phosphorylation, acetylation, SUMOylation, succinylation, ubiquitination, methylation, glycosylation, etc. Different types of PTMs have different impacts on Tau's structure and function. Phosphorylation, acetylation, SUMOylation, and succinylation mainly have negative effects, promoting Tau aggregation. Interestingly, ubiquitination, methylation, and glycosylation can have both positive and negative effects in regulating the level of Tau phosphorylation, degradation, aggregation, and microtubule stability. Drug clinical studies on PTM‐regulated Tau are currently very scarce, and most research is still in the basic research stage.

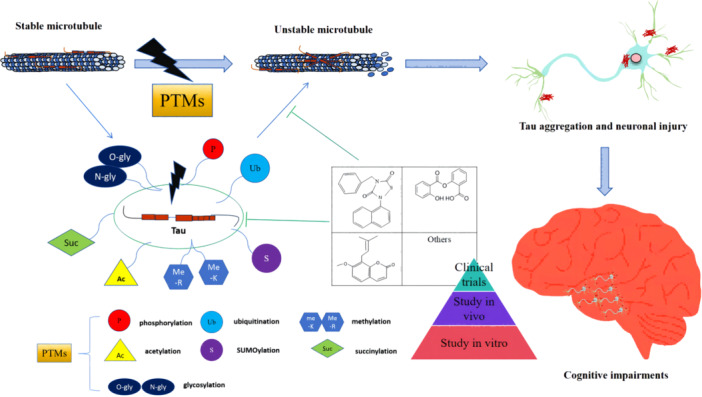

## 1 INTRODUCTION

Alzheimer's disease (AD) is a neurodegenerative disorder characterized by progressive cognitive impairment and behavioral abnormalities, occurring in the elderly and pre‐elderly. It accounts for 70%–80% of all dementia cases. Clinical manifestations include memory loss, impaired spatial abilities, impaired abstract thinking and calculation skills, as well as personality and behavioral changes. As of 2016, there were approximately 50 million dementia patients worldwide, and this number is expected to reach 152 million by 2050, posing a significant economic burden on families and society.^1^

The main pathological features of AD are the formation of amyloid plaques composed of β‐amyloid peptide (Aβ) and neurofibrillary tangles (NFTs) formed by abnormally modified Tau protein in neurons. Synaptic damage and neuronal loss are the primary factors leading to cognitive decline. In the past, the development of AD therapies has mainly focused on Aβ. However, the majority of drugs developed for Aβ‐targeted treatments have ended in failure.^2^ Given that Tau accumulates approximately 10 years earlier than Aβ, and abnormal phosphorylated Tau (p‐Tau) on microtubules increases Aβ production,^3^ treatments targeting Tau are gradually becoming the focus for AD. In the Tau triple transgenic AD (3×Tg‐AD) mouse model, passive immunotherapy targeting Tau not only inhibits abnormal Tau aggregation but also reduces Aβ deposition.^4^ In addition, postsynaptic mitogen‐activated protein kinase p38γ mediates Tau T205 site‐specific phosphorylation, interfering with the formation of Aβ‐induced postsynaptic excitotoxic signaling complexes, thereby protecting neurons from Aβ excitotoxicity.^5^

Posttranslational modifications (PTMs) play a crucial role in regulating Tau function and structure, but an imbalance between PTMs may lead to abnormal Tau function and aggregation. Aberrant phosphorylation of Tau results in the formation and accumulation of NFTs in AD. In addition to phosphorylation, Tau undergoes other forms of PTMs, including acetylation, ubiquitination, glycosylation, glycation, small ubiquitin‐like modifier mediated modification (SUMOylation), methylation, succinylation, etc.^6^ All of these PTMs can all affect the function and structure of Tau. Targeting any one of these modifications alone or in combination has the potential to prevent Tau aggregation and restore normal protein function. This review primarily discusses the impact of PTMs on Tau and the progress in drug research based on PTMs, providing new insights for the diagnosis and treatment of AD.

## 2 THE STRUCTURE AND FUNCTION OF TAU

Tau is a microtubule‐associated protein (MAP) encoded by the microtubule‐associated protein tau (MAPT) gene on chromosome 17 and is mainly present in the Neuronal axons.^7^ Selective splicing of exons 2, 3, and 10 generates six isoforms of Tau: 0N/3R, 0N/4R, 1N/3R, 1N/4R, 2N/3R, and 2N/4R. Tau consists of four main domains: the N‐terminal domain, the proline‐rich domain, the microtubule‐binding domains (MBDs), and the C‐terminal domain.^8^ Tau is a key protein for microtubule stabilization in neuronal axons and also plays a crucial role as a synaptic protein in accelerating dendritic spine formation, dendritic extension, and synaptic plasticity.^9^ However, Tau is also an intrinsically disordered protein that accumulates and aggregates due to various abnormal PTMs, forming toxic intracellular paired helical filaments (PHFs) and NFTs.^10^ NFTs first appear in the entorhinal cortex and hippocampus, spreading to the limbic system and association cortices as the disease progresses. The number of NFTs in AD patients is positively correlated with the severity of dementia, supporting the central role of Tau in AD.^7^

## 3 PTMS AND TAU

**Figure 1:**
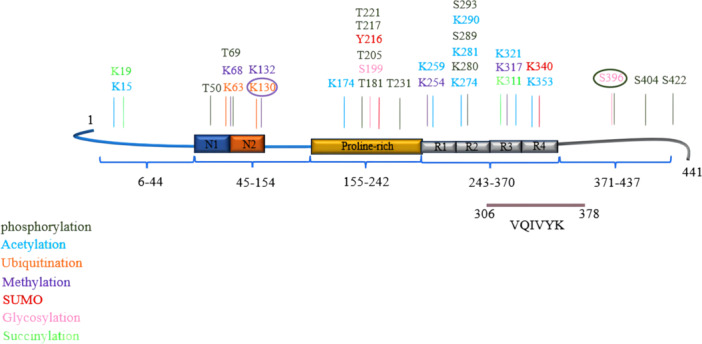
The amino acid sites of different PTMs at Tau. Tau has a total of 441 amino acid sites, which can undergo a variety of PTMs (Different colors represent different PTMs, and circles represent two kinds of modifications at this site). PTMs, posttranslational modifications. [Color figure can be viewed at wileyonlinelibrary.com]

It is acknowledged that PTMs regulate the function, levels, and aggregation of Tau, and are critical factors for Tau stability.^11^ PTMs refer to modifications that occur on proteins after translation by ribosomes or after their folding and localization have been completed. PTMs alter the charge and hydrophobicity of proteins, leading to structural changes that can affect protein function, protein–protein interactions, and aggregation.^12^ Tau is a typical naturally unfolded protein that, due to the lack of a tertiary structure, can undergo numerous PTM modifications,^13^ involving the addition of small chemical groups or peptides on different Tau side chains, such as phosphorylation on serine (S), threonine (T), or tyrosine(Y), acetylation, ubiquitination, SUMOylation, and glycosylation on lysine (K), or methylation on arginine (R).^11^ Wesseling et al. identified 55 phosphorylation sites, 17 ubiquitination sites, 19 acetylation sites, and 4 methylation sites on Tau through analysis of postmortem brain tissue from AD patients.^14^ These PTMs play a critical role in regulating Tau microtubule interactions, localization, degradation, and aggregation, which can affect Tau aggregation propensity or function. Therefore, PTMs may be key contributing factors to the onset of AD (Figure 1).

## 4 TYPES OF PTMS IN TAU AND TREATMENTS

### 4.1 Phosphorylation and related treatments

**Figure 2:**
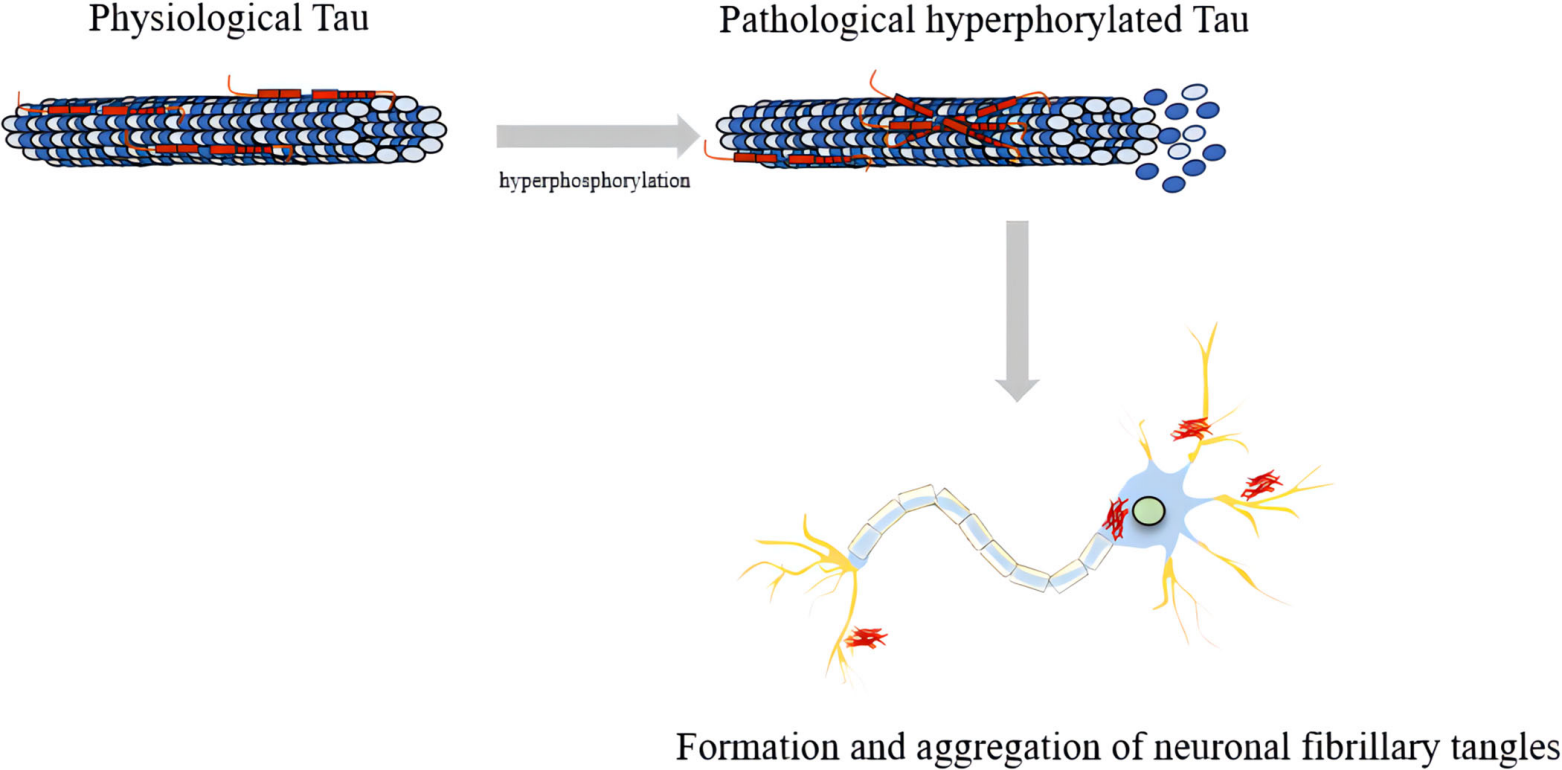
The hyperphorylation of Tau leads to the neurofibrillary tangles in neurons. Tau is a microtubule‐associated protein that binds to microtubules to stabilize microtubules. When Tau is hyperphorylated, the assembly activity of microtubules is destroyed, and neuronal tangles are formed and deposited in neurons. [Color figure can be viewed at wileyonlinelibrary.com]

Phosphorylation involves reversibly adding a phosphate group to the polar group of S, T, or Y residues. Phosphorylation is necessary for the binding of Tau to microtubules, however, excessive phosphorylation of Tau leads to its separation from microtubules and aggregation^15^ (Figure 2). In early studies, it was found that aggregated Tau isolated from AD brains was three to four times higher compared to healthy controls.^16^ Phosphorylation of Tau protein in the proline‐rich domain disrupts its microtubule assembly activity and slightly increases Tau self‐aggregation propensity. Phosphorylation of the C‐terminal domain of Tau significantly promotes its self‐aggregation.^17^ T50, T69, T181, and T205 are key sites regulating multi‐site phosphorylation levels of human Tau protein. Inhibiting the phosphorylation of these key sites may disrupt the inherent enhancement mechanism of Tau phosphorylation and reduce pathological Tau levels.^18^ It was reported that through all‐atom molecular dynamics simulation techniques that phosphorylation at the S289 and S293 sites not only reduces the binding affinity between Tau repeat 2 (R2) and microtubules but also promotes Tau aggregation by enhancing the transition of monomeric peptides into a helical structure.^19^ In AD associated with autosomal dominant mutations, excessive phosphorylation of Tau in cerebrospinal fluid (CSF) occurs very early and exhibits site‐specific changes at different stages of the disease. The ratio of p‐Tau217/T‐217 and p‐Tau181/T‐181 significantly increases with the appearance of Aβ pathology and decreases significantly in the clinical late stage. Levels of p‐Tau205 and T‐Tau increase in the late stages of AD and continue to rise as the disease progresses.^20^ In the preclinical stages of AD, CSF p‐Tau217 and p‐Tau231 can differentiate whether patients have Aβ deposition.^21^ CSF p‐Tau217 is better than p‐Tau181 in distinguishing Tau from other neurodegenerative diseases.^22^ Blood tests have the convenient and cost‐effective advantage, and blood p‐Tau181, p‐Tau217, and p‐Tau231 can serve as biomarkers for the diagnosis and prognosis of AD. Therefore, early screening of blood p‐Tau may significantly reduce the application of positron emission tomography (PET) scans or lumbar punctures clinically.^23^, ^24^

The phosphorylation status of Tau is regulated by Tau kinases and phosphatases, among which kinases include glycogen synthase kinase 3 beta (GSK‐3β), cyclin‐dependent kinase 5 (CDK‐5), and cAMP‐dependent protein kinase A (PKA).^25^ GSK‐3β can phosphorylate at least 26 residues of Tau, however, excessive activation of GSK‐3β leads to hyperphosphorylation of Tau, microtubule destruction, and neuronal apoptosis.^26^ There are two types of GSK‐3β inhibitors, thiazolidinedione derivatives (Tideglusib) and lithium salts, which have been evaluated in clinical studies. Tideglusib reduces hyperphosphorylation of Tau, Aβ deposition, neuronal loss, and improves learning and memory in AD animal models.^27^ Phase II trials of Tideglusib indicate that short‐term (26 weeks) use is within a safe range, but did not demonstrate significant clinical benefits.^28^ Lithium salts are mainly used to treat bipolar disorder. Meta‐analyses of cohort and case‐control studies suggest an association between lithium salts and reduced risk or severity of dementia. Another meta‐analysis of a three‐arm randomized controlled phase II study also indicates that lithium salts improve cognitive abilities in patients with mild cognitive impairment (MCI) and AD, with lithium salts showing superior efficacy to aducanumab and at a lower cost.^29^, ^30^

ANAVEX2‐73, an amino‐tetrahydrofuran derivative (a muscarinic receptor agonist and σ₁ receptor agonist), possesses anti‐amnesic and neuroprotective properties. This compound has been found to impair the activation of Aβ‐mediated GSK‐3β and protein kinase B(Akt/PKB) and block or reduce phosphorylation of different Tau epitopes in AD mice.^31^

According to traditional Chinese medicine records, ginsenoside Rg1 has the effect of alleviating cognitive impairment. Okadaic acid (OA) injection induces high expression of p‐Tau in the CA3 and CA1 regions of the parietal cortex, which can be reversed by ginsenoside Rg1 and donepezil. Besides, the expression of GSK‐3β in AD rats treated with ginsenoside Rg1 or donepezil was significantly lower than that of untreated AD model group rats, indicating that ginsenoside Rg1 may alleviate cognitive impairment and improve pathological changes in AD by inhibiting the phosphorylation.^32^ Similarly, a study reported that ginsenoside Rg1 can reduce isoflurane‐induced hyperphosphorylation of Tau in dementia rats by inhibiting the expression of GSK‐3β.^33^ The above results indicate that ginsenoside Rg1 can reduce hyperphosphorylation of Tau in AD by inhibiting the GSK‐3β signaling pathway.

In a study by Ni et al., 117 6‐month‐old APP/PS1 mice were treated with Osthole extract for 42 days. The results showed that, compared to the model group, the expression of p‐Tau (S202) was significantly reduced in the Osthole extract group. In addition, the expression levels of phosphoinositide 3‐kinase (PI3K), p‐Akt/Akt, and p‐GSK3β/GSK3β were significantly increased. These results suggest that Osthole extract may regulate the activity of the PI3K/AKT/GSK‐3β signaling pathway, thereby reducing the hyperphosphorylation of Tau protein.^34^

Protein phosphatase 2A (PP2A) accounts for over 70% of Tau phosphatase.^35^ In AD, the activity of PP2A is significantly reduced. Sodium selenate, a negatively charged anionic compound, activates PP2A in vivo and in vitro and can reverse memory deficits and reduce Tau phosphorylation levels in AD animal models.^35^ A phase IIa trial of sodium selenate (30 mg/day) in 40 patients with mild to moderate AD confirmed the safety and tolerability of the drug.^36^

The Y oligopeptide inhibitor KYP‐2047 reduces Tau levels and slows down cognitive decline in AD animal models by activating PP2A.^37^ Other drugs that act by blocking the inhibitory effect on PP2A include memantine, apolipoprotein mimetic COG112, and sphingosine‐1‐phosphate receptor agonist FTY720.^38^ SEW2871 is a more selective sphingosine 1‐phosphate receptor agonist than FTY720, which can reduce the p‐Tau（S262）in rat hippocampal slices.^39^ PTI‐125 is a small molecule candidate drug. Wang et al. found that in 13 patients with mild to moderate AD who were orally administered the drug, the drug can inhibit Aβ_‐42_‐induced Tau hyperphosphorylation through α‐7 nicotinic acetylcholine receptor signaling, reduce p‐Tau181 levels, and slow the rate of neurodegeneration by 40%.^40^

When applied to Tau‐P301S transgenic mice, hesperidin is reported to reduce pathological Tau levels by increasing PP2A levels, thereby regulating Tau protein hyperphosphorylation. Additionally, hesperidin can also downregulate the nuclear transcription factor kappa B (NF‐kB) pathway to inhibit gliosis and neuroinflammation, prevent microglial synaptic engulfment, rescue synaptic loss in the mouse brain, and significantly improve cognitive abilities.^41^

Banqiao *Codonopsis Pilosula* (BCP) can reduce the demethylation and phosphorylation levels of PP2A, leading to an upregulation of PP2A activity. Therefore, by reducing the phosphorylation levels of Tau protein at phosphorylation sites T181, T231, S396, and S404, excessive phosphorylation of Tau protein can be suppressed. In addition, BCP increases the expression levels of synaptophysin‐1, N‐methyl‐d‐aspartate‐2B(NMDA‐2B), and synaptophysin in hippocampal tissue by upregulating PP2A activity, promoting neuronal repair.^42^

### 4.2 Acetylation and related treatments

Protein acetylation is one of the major PTMs in eukaryotes, referring to the transfer of an acetyl group from acetyl coenzyme A (acetyl‐CoA) to specific sites on the peptide chain. Acetylation of Tau at K280 leads to the formation of toxic oligomers and short protofibrils, significantly increasing the aggregation rate of Tau and impairing its microtubule assembly ability.^43^ Acetylation of Tau K280 is associated with the early stages of AD and is believed to occur before Tau hyperphosphorylation.^43^ In addition, acetylation of Tau at K174 also occurs in the early stages of AD and is a critical determinant for maintaining the stability of mouse Tau, while acetylation at K274 and K281 appears in the later stages of AD progression.^44^ Liu et al. simulated Tau K274 and K281 acetylation in vitro, resulting in reduced mitochondrial biogenesis, decreased expression levels of mitochondrial fusion proteins, exacerbated mitochondrial dysfunction, and thereby worsened cognitive impairments.^45^ Furthermore, acetylation sites on Tau at the KXGS motif (K259, K290, K321, K353) inhibit phosphorylation within the motif and prevent Tau aggregation.^46^ Zhou et al. found that Tau overexpression significantly promotes the acetylation of GSK‐3β at K15 and inhibits its ubiquitination in vitro. Acetylation at K15, in turn, increases Tau phosphorylation levels, leading to synaptic dysfunction and memory deficits, forming a vicious cycle. Competitive inhibition of GSK‐3β acetylation at K15 can significantly improve cognitive impairments and AD‐like pathology in 3×Tg‐AD mice.^44^ Depending on the specific modification site, Tau acetylation can either promote or inhibit Tau aggregation.

Salsalate is a prodrug of salicylate and can inhibit p300‐induced Tau acetylation. Min et al. demonstrated through AD animal experiments that salsalate can inhibit p300, thereby reducing K174 acetylation and levels of AT8‐positive p‐Tau.^47^ Histone deacetylase 6 (HDAC6) is increased in the brains of AD patients and is associated with deacetylation of Tau and the formation of Tau aggregates. Deacetylation of the K321 residue promotes phosphorylation of the S324 residue, thereby facilitating excessive phosphorylation of Tau by GSK‐3β. Tubastatin A or ACY‐1215 selectively inhibit HDAC6, thereby reducing excessive phosphorylation of Tau by inhibiting the protein kinase B/GSK‐3β (Akt/GSK3‐β) signaling pathway. This can alleviate cognitive deficits in APPswe/PS1^ΔE9^ (PAP) mice.^48^ Furthermore, Choi et al. utilized brain tissues from AD patients and AD‐like pathology APP & Tau mice and found that the HDAC6 inhibitor CKD‐504 can efficiently penetrate the blood–brain barrier. It degrades pathological Tau and reduces synaptic defects.^49^

### 4.3 Ubiquitination and related treatments

During the ubiquitination process, the ubiquitin‐conjugating enzyme (E2)/ubiquitin ligase (E3) ligase complex can attach a single ubiquitin to a single (monoubiquitination) or multiple Kresidues (polyubiquitination) on the target protein. Tau, being a protein rich in Kresidues, is highly sensitive to ubiquitination. Hendrik et al. identified 28 ubiquitination sites in postmortem AD brain tissue using immunoprecipitation, with 16 of them located on the MBD that forms the fibrillar core.^50^ Ubiquitin‐specific peptidase 11 (USP11) expression is higher in the brains of females than males and is closely related to female Tau pathology. This may be one of the reasons why the incidence of AD is higher in females than males. USP11 promotes pathological Tau aggregation by deubiquitinating with K281, thereby facilitating Tau pathology formation.^51^ Ubiquitination of the C‐terminus of Tau in AD patients can stabilize the protofibril interface formed between Tau filaments. Mono‐ and polyubiquitin chains constitute key components of the fibrillar core.^52^ Some studies suggest that ubiquitination of Tau enhances its ability to be cleared by the proteasome or the lysosome‐autophagy system.^53^ However, ubiquitinated Tau oligomers may significantly impair the function of the proteasome.^54^ Soluble Tau oligomers (sTauO) are the most neurotoxic form of Tau. Puangmalai et al. identified 11 ubiquitination sites on sTauO of AD patients using mass spectrometry, among which ubiquitination at K63 associated sTauO accumulated at high levels in the brains of AD patients.^55^ Ubiquitination of Tau can maintain microtubule stability, inhibit Tau aggregation, but ubiquitination of sTauO leads to increased formation and secretion of sTauO.

Based on this, ubiquitination may protect neurons from Tau oligomers toxicity by promoting the formation of insoluble NFTs. So far, only one deubiquitinase of Tau, OTU domain ubiquitin aldehyde binding 1 (OTUB1), has been identified in mice, and it is currently being developed for cancer treatment, which may represent a new therapeutic approach for AD.^56^

TH006 is a chimeric complex that can simultaneously bind Tau and E3 ligases, promoting Tau polyubiquitination, specifically inducing Tau degradation in 3×Tg‐AD mice and N2a cells overexpressing Tau, and reducing Aβ‐induced cytotoxic effects.^57^ Similar chimeric C004019 has similar effects and improves cognitive function in AD mouse models. Subcutaneous injection of C004019 significantly reduces Tau levels in the brains of wild‐type, hTau transgenic, and 3×Tg‐AD mice, as well as improves synaptic and cognitive function.^58^ Proteolysis‐targeting chimaeras (PROTACs) recruit substrate adapter proteins of Kelch‐like ECH‐associated protein 1 (Keap1) ubi ‐quitin E3 ligase to bind to Tau, thereby degrading Tau in cells through the polyubiquitination pathway.^59^ Now PROTACs have become a pathway for discovering new drugs of AD.

### 4.4 Methylation and related treatments

Methylation refers to the addition of one or more methyl groups to the side chains of K or R. Tau can undergo monomethylation or dimethylation, while trimethylation has not been reported in Tau. Methylation of K is a physiological PTM of Tau, and with age and disease progression, it transitions from dimethylation to monomethylation.^60^ Bichmann et al. reported that the methyltransferase SET domain containing 7 (SETD7) is a novel K methyltransferase that catalyzes monomethylation of Tau at K130 and K132. Tau methylation levels increase with AD progression, and SETD7 is abundant in soluble brain extracts and preferentially localized to the cytoplasmic and nuclear fractions, indicating that K methylation may regulate the subcellular localization of Tau.^61^ In AD, the hypomethylation status of neurons is associated with Tau aggregation, increased expression of senescence markers, and Aβ deposition.^62^ However, residues like K254 can undergo methylation and ubiquitination, with methylation potentially inhibiting the degradation of Tau protein by impeding proteasomal clearance.^63^ Additionally, methylation of Tau at K317 affects its binding to microtubules through non‐phosphorylation pathways, promoting Tau aggregation. However, K317 methylation may also promote neuronal maturation by facilitating dendritic elongation.^64^ As AD progresses in patients, methylation gradually decreases, leading to increased Tau aggregation. However, methylation at specific sites may compete with ubiquitination and impede the degradation of Tau. The hypomethylation status of neurons is associated with Tau aggregation, increased expression of senescence markers, and Aβ deposition. Therefore, increasing neuronal methylation may be beneficial for the treatment of AD.

Natural compounds that can regulate DNA methylation status may provide a new approach for AD treatment. Epigallocatechin‐3‐gallate (EGCG), a compound found in green tea, competitively inhibits DNA methyltransferase 1 (DNMT1) and reactivates genes silenced by DNMT1‐mediated methylation.^65^, ^66^ Other natural small molecules, such as naringin, apigenin, quercetin, curcumin, and genistein, have a certain impact on DNA methylation.^67^ Excessive phosphorylation of Tau is associated with decreased methylation of PP2A,^68^ and folic acid can reduce Tau phosphorylation by regulating the methylation of PP2A in diabetic mice.^69^

S‐adenosylmethionine (SAMe) is the active form of the amino acid methionine and is a major metabolic product in various pathways critical to neuronal homeostasis. As a methyl donor, SAMe affects gene expression by influencing DNA methylation, leading to the synthesis of various products, including neurotransmitters and phosphatases.^70^

### 4.5 SUMOylation and related treatments

SUMOylation refers to the attachment of small ubiquitin‐like modifier (SUMO) proteins to target protein K residues and is a reversible PTM. There are currently three major homologs of SUMO: SUMO‐1, SUMO‐2, and SUMO‐3. Overexpression of SUMO‐1 leads to impaired synaptic function, decreased dendritic spine density, and significant memory deficits.^71^ Enhanced SUMO‐1 immunoreactivity is detected in cortical Tau aggregates in the brains of AD patients.^72^ SUMOylation at Tau K340 stimulates Tau phosphorylation and inhibits proteasomal degradation, promoting Tau aggregation.^73^ SET is an inhibitor of PP2A. In AD mouse models, SET K68 is SUMOylated, which inhibits PP2A activity and leads to excessive Tau phosphorylation.^74^ Recent studies have shown that SUMOylation of GSK‐3β can induce phosphorylation of the kinase at Y216 and subsequent activation,^75^ suggesting that SUMOylation of protein kinases or phosphatases may also lead to increased Tau phosphorylation. Therefore, SUMOylation of GSK‐3β may be another promising target for AD treatment.

### 4.6 Glycosylation and related treatments

Glycosylation is the process by which a sugar moiety is transferred to a protein by a glycosyltransferase, forming a glycosidic bond with an amino acid residue on the protein. Depending on the position of the glycosidic bond, glycosylation can be classified as O‐ or N‐glycosylation. In the brains of AD patients, O‐glycosylation is significantly reduced. O‐glycosylation of Tau reduces aggregation by preventing its phosphorylation at the same residues.^76^ In HEK293‐Tau‐BIFC cells, the dynamic changes in Tau structure can be observed. Inhibiting glycosyltransferase leads to increased phosphorylation levels of Tau at S199 or S396, which enhances Tau aggregation.^77^ Histone deacetylase Sirtuin type 1 (SIRT1) deacetylates cyclic adenosine monophosphate (cAMP) response element‐binding protein (CREB) to suppress O‐linked N‐acetylglucosamine transferase (OGT) expression, thereby reducing Tau glycosylation and increasing phosphorylation of Tau at specific sites.^78^ N‐glycosylation is present in the brains of AD patients but has not been found in healthy brains. In AD patients, Tau is N‐glycosylated, which helps maintain and stabilize the PHF structure, reducing Tau aggregation propensity.^79^ Previous studies have suggested that glycosylation can upregulate kinase activity, leading to Tau hyperphosphorylation.^80^ However, numerous studies also suggest that glycosylation reduces Tau phosphorylation and decreases Tau aggregation.^81^

Studies in vivo have shown that modulating glycosylation levels can be an effective target for treating AD. The small molecule Thiamet‐G inhibits glycosidases, increasing glycosylation and reducing the phosphorylation levels of Tau at T231, S396, and S422. Long‐term treatment with Thiamet‐G in rTg4510 mice almost completely inhibits glycosidases, reducing pathological Tau in the mouse brain and total Tau levels in the CSF.^82^, ^83^

### 4.7 Succinylation and related treatments

Succinylation, a unique and rarely studied type of PTM, is common in mitochondria and helps maintain normal metabolic function.^84^ Cellular metabolism is affected in both AD and other dementia patients, and changes in cellular metabolism lead to a decrease in the succinylation of multiple mitochondrial proteins and an increase in the succinylation of Tau in AD. As the self‐assembling sequence within Tau, PHF6 (residues 306‐311) and PHF6* (residues 275‐280) are nucleation sequences that initiate the process of Tau aggregation.^85^ Yang et al. examined 10 AD brain tissue samples, in which nine samples were detected with Tau succinylation at K311 in the PHF6 hexapeptide 306VQIVYK311, which was not found in all control samples. Tau K311 succinylation significantly reduced the binding affinity of Tau to microtubule proteins and markedly accelerated the aggregation of PHF6.^86^ Tau K19 promotes microtubule assembly, and after succinylation by succinyl coenzyme A, it completely inhibits its microtubule assembly activity.^84^ Tau succinylation may provide new therapeutic targets for the prevention and/or treatment of AD and related pathologies.^87^ As the mechanism of succinylation is still under investigation, there is a need to develop new therapies and related drugs based on the new succinylation and desuccinylation molecular mechanisms for more definitive evidence.

## 5 SUMMARY AND OUTLOOK

This review discussed the effects of various types of PTMs on Tau and the current status of drug development related to this. Tau is one of the primary pathological features of AD. Tau can undergo various PTMs, and different types of modifications have different impacts on Tau's structure and function. Hyperphosphorylation, acetylation, SUMOylation, and succinylation mainly have negative effects, promoting Tau aggregation. On the other hand, ubiquitination, methylation, glycosylation, etc., can have both positive and negative effects, promoting/reducing Tau phosphorylation levels, inhibiting/promoting its degradation, affecting Tau aggregation, and influencing microtubule stability. Furthermore, various PTMs have overlapping sites, thereby influencing each other. Based on research on the different types of PTMs and their mechanisms of action in regulating Tau aggregation, many drugs are currently being developed. However, the current focus is mainly on Tau phosphorylation and ubiquitination. As research continues, other PTMs such as methylation, glycosylation, acetylation, SUMOylation, and succinylation are also receiving more attention. Drug clinical studies on PTM‐regulated Tau are currently very scarce, and most research is still in the basic research stage (Table 1). Finding effective drugs for AD is still a very challenging direction in disease treatment, but in‐depth research on the various PTM regulatory mechanisms of Tau and the development of corresponding drugs may become an important therapeutic strategy for treating AD.

**Table 1:** Drugs developed based on the regulatory mechanism of Tau PTMs.

Drugs	Molecule	Molecular weight	Subjects or models	Mechanism	References
Tideglusib	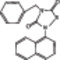	334.39	AD patients	Inhibit the activation of GSK‐3β and reduce p‐Tau	[28]
Lithium	Li^+^	6.94	AD patients	Inhibit the activation of GSK‐3β and reduce p‐Tau	[29, 30]
ANAVEX_2‐73_	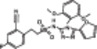	497.50	A*β*_25–35_ induced AD mice	A mixed muscarinic and σ₁ receptor agonist: reduce p‐GSK‐3β on Yand p‐AKT.	[31]
Gensenoside Rg1	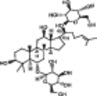	801.013	OA‐induced AD rats' brain slice	Upregulate the expression of PP2A to inhibit the GSK‐3β pathway.	[32, 33]
Osthole	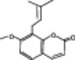	244.29	APP/PS1 mice	Downregulate the level of p‐Tau (S202), modulate the PI3K/AKT/GSK‐3β signaling pathway	[34]
Sodium selenate	Na2SeO4	188.937	AD patients	Activate PP2A	[35, 36]
KYP‐2047	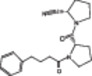	339.43	PS19 tau transgenic mice: HEK‐293 cells/N2a cells/Human iPSC derived neurons (P301L/Tau‐A152T)	A prolyl oligopeptidase inhibitor: activate PP2A	[37]
SEW2871	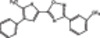	440.362	Rat hippocampal slices	A sphingosine‐1‐phosphate receptor 1 agonist: reduce p‐Tau (S262)	[39]
PTI‐125	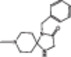	295.81	Mild to moderate AD patients	Inhibit nicotinic acetylcholine receptor signaling, reduce p‐Tau181 levels	[40]
Rutin	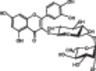	610.52	Tau‐P301S mice	Regulated p‐Tau by increasing PP2A levels, inhibited glial proliferation and neuroinflammation by downregulating the NF‐kB pathway and prevented microglial synaptic phagocytosis	[41]
Banqiao *Codonopsis Pilosula*	–	–	OA‐induced AD rats	Upregulated PP2A activity and reduced p‐Tau (T181, pT231, pS396 and pS404)	[42]
Salsalate	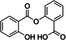	258.23	PS19 transgenic mouse model of FTD	Inhibited tau acetylation at K174, reduced total Tau levels	[47]
Tubastatin A/ACY‐1215	–	–	APP^swe^/PS1^ΔE9^ (PAP) double‐transgenic mice (C57BL/6 J)	Inhibition of Akt/GSK3β signal pathway reduces Tau hyperphosphorylation	[48]
CKD‐504	–	–	ADLP^APT^ mice	HDAC6 inhibitor: regulating the interaction between Tau, chaperone proteins and E3 ligases, increases the acetylation of Tau, chaperone proteins, and E3 ligases, thereby accelerating the degradation of Tau by proteasomes	[49]
TH006	–	–	3×Tg‐AD mice, N2a cells overexpressing Tau	Promoting Tau degradation by increasing polyubiquitination	[57]
C004019	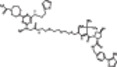	1035.29	Cells: HEK293/SH‐SY5Y(hTau) Mice: hTau‐transgenic and 3xTg‐AD mice	Enhanced ubiquitin‐proteasome‐dependent protein degradation	[58]
PROTACs	–	–	SH‐SY5Y cells transfected with Tau	Recruitment of substrate adaptor proteins for Keap1 ubiquitin E3 ligase to interact with Tau, thereby promoting intracellular Tau degradation through the polyubiquitination pathway.	[59]
Folic acid	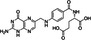	441.397	Diabetes mice	Increase PP2A methylation and reduce p‐Tau	[69]
Thiamet‐G	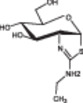	248.299	PC12 cells, rTg4510 mice	inhibits glycosidase, reduced p‐Tau(T231, S396, S422) and Tau levels.	[82, 83]

Abbreviations: AD, Alzheimer's Disease; phosphoinositide 3‐kinase (PI3K); Akt, protein kinase B; FTD, frontotemporal dementia; GSK‐3β, glycogen synthase kinase 3 beta; HDAC6, Histone deacetylase 6; OA, Okadaic acid; PP2A, protein phosphatase 2A; S, serine; T, threonine; Y, tyrosine.

## AUTHOR CONTRIBUTIONS

Yong Luo and Nanqu Huang contributed to the main ideas of this review, leading to the submission of the paper. Xin Li, Zhisheng Ba contributed to the organizational thinking, writing and revision of the paper, finalized the manuscript, and approved the final version for review. Juan Huang, Jianhua Chen and Jinyu Jiang participated in the production and modification of some charts and provided suggestions for some modifications of the paper. All authors have read and approved the final manuscript.

## CONFLICT OF INTEREST STATEMENT

The authors declare no conflict of interest.

## ETHICS STATEMENT

Not applicable.

## Data Availability

Not applicable as no new data are generated in this review article.
